# Lepidopterism: Case Report and Review of the Literature

**DOI:** 10.7759/cureus.6567

**Published:** 2020-01-05

**Authors:** Enrique Konstat-Korzenny, Alexander Yudovich, Dan Morgenstern-Kaplan

**Affiliations:** 1 Facultad de Ciencias de la Salud, Universidad Anáhuac Mexico, Mexico City, MEX; 2 General Pediatrics, Pediatric Associates, Houston, USA

**Keywords:** pediatrics, dermatology, caterpillar, lepidopterism, sting, environment toxicology, venom

## Abstract

Lepidopterism is a term that refers to a spectrum of medical conditions, typically involving the skin, that result from contact with the adult or larval forms of certain butterflies and moths. There are more than 165,000 species of these insects, however, only about 12 species are known to harm humans, most commonly in the form of contact dermatitis. Among these species, the Megalopyge opercularis, commonly called the "Puss Caterpillar", is known to cause a painful and pruritic cutaneous reaction when its venom encounters the skin. Although caterpillar stings are not a common etiology of dermatological rashes, physicians must perform a detailed history and have a high degree of suspicion to arrive to the correct diagnosis and avoid unnecessary medications and therapeutics. We present a case of a 14-month-old boy who presents to the pediatric clinic with a unilateral red rash on the anterior aspect of the left leg, from the distal thigh to the shin. The parents report that the boy was sitting in the park, when he suddenly started to cry. They state that the rash began to spread and that red marks are growing. The patient's parents brought the caterpillar specimen in a bag, clinching the diagnosis. The patient was treated with antihistamine drugs for symptom relief and was recommended to wash thoroughly the area with soap and water. The patient returned to the clinic four days later and the rash had resolved. When encountering an acute onset rash in a patient with recent exposure to nature environments and other open spaces with trees, lepidopterism should be considered in the differential diagnosis and promptly treated. Furthermore, the physician must educate the patients on how to avoid exposure, and special care should be implemented with asthmatic and atopic patients, because although rare, anaphylactic reactions to these stings have been reported.

## Introduction

Lepidopterism is a term that refers to a spectrum of medical conditions in humans that usually involve the skin and result from contact with the adult or larval forms of certain butterflies and moths [1,2]. Caterpillars are the worm-like, larval forms of butterflies and moths of the insect order Lepidoptera. There are more than 165,000 species of these insects, but only about 12 species are known to be harmful to humans, most commonly causing contact dermatitis [3].

Among these species, the Megalopyge opercularis, commonly called the "Puss Caterpillar", is known to cause a painful and pruritic cutaneous reaction that occurs when the caterpillar comes in contact with the skin and injects the venom located in its spines [4]. These caterpillars are commonly found in trees in the Southern Atlantic Coast, as well as the Gulf Coast, including Louisiana, Alabama, Mississippi and Texas [5,6]. The peak incidence of the stings occurs in the summer months of June and July. In the present work, we discuss the case of a 14-month-old male patient with an acute onset rash caused by a Megalopyge opercularis sting (lepidopterism) in Texas, USA.

## Case presentation

A 14-month-old boy presents to a pediatric clinic with a two-hour history of a unilateral red rash on the anterior aspect of the left lower extremity, from the distal thigh to the shin. The parents endorse that the rash is spreading and red marks are growing. The patient is up to date with all immunizations for his age, has no known drug allergies, takes no daily medication and does not have a relevant medical history.

**Figure 1:**
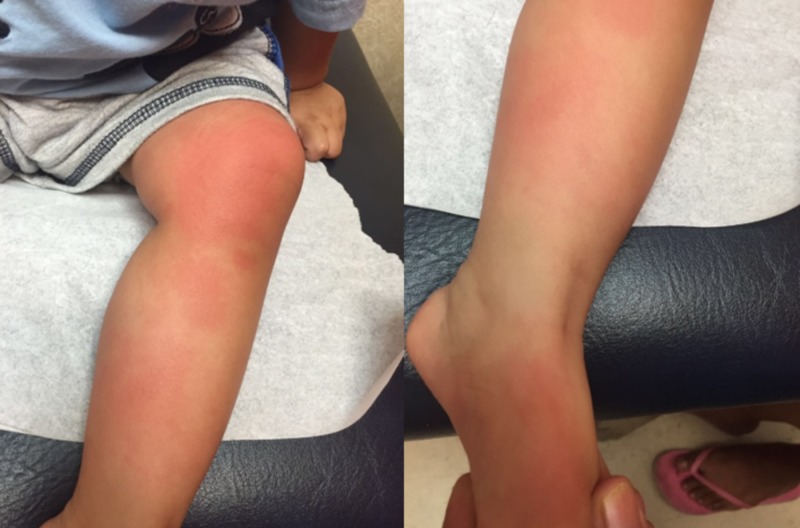
Rash on the lower extremity caused by the Megalopyge opercularis sting

**Figure 2:**
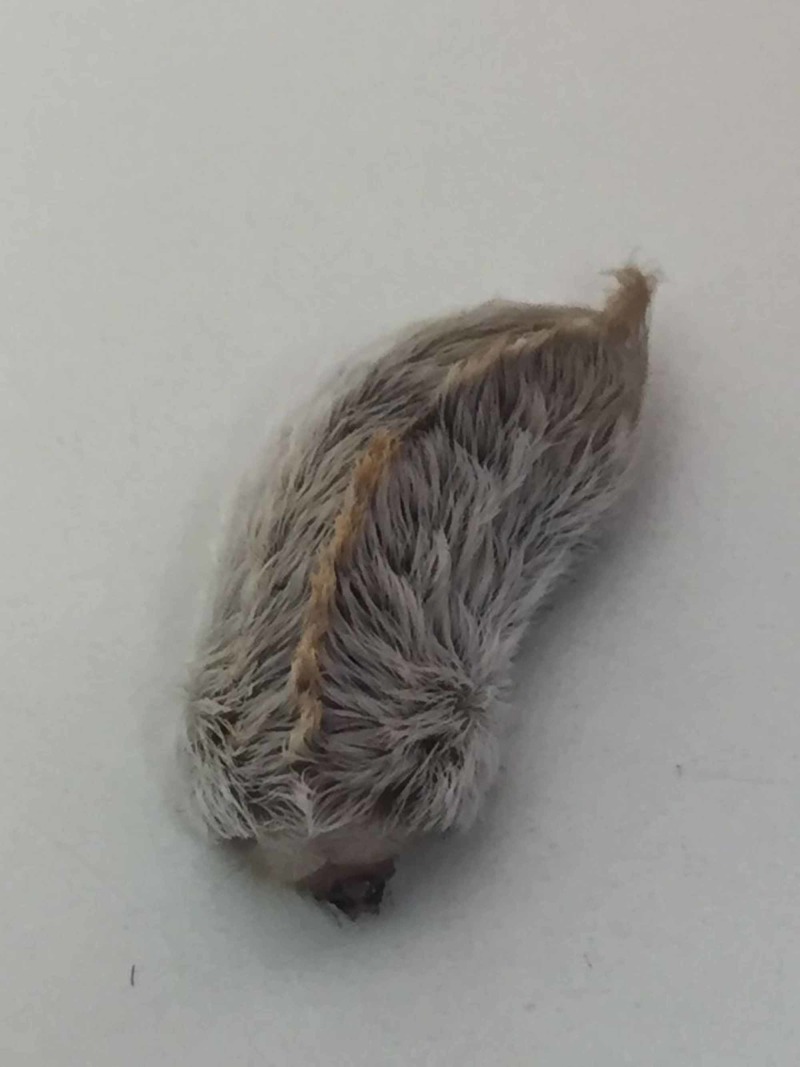
Megalopyge opercularis (also known as Puss Caterpillar) specimen brought to the office by the patient's family

The parents report that the boy was sitting in the park, when he suddenly started to cry. As the parents approached him, they saw an "insect" crawling on his leg. The parents caught the "insect" and brought it to the office for the pediatrician to view. Examination revealed a well appearing child in no apparent distress, his temperature was 37.6 °C, chest auscultation revealed clear lungs with no wheezing or rhonchi, regular heart rhythm and respiration rate. His left leg presented a blanching red rash in discrete patches and plaques that was not warm to palpation (Figure 1). The insect brought in was a "Puss Caterpillar" (Figure 2). There were no visible spines or setae in the patient's skin to be removed.

**Figure 3:**
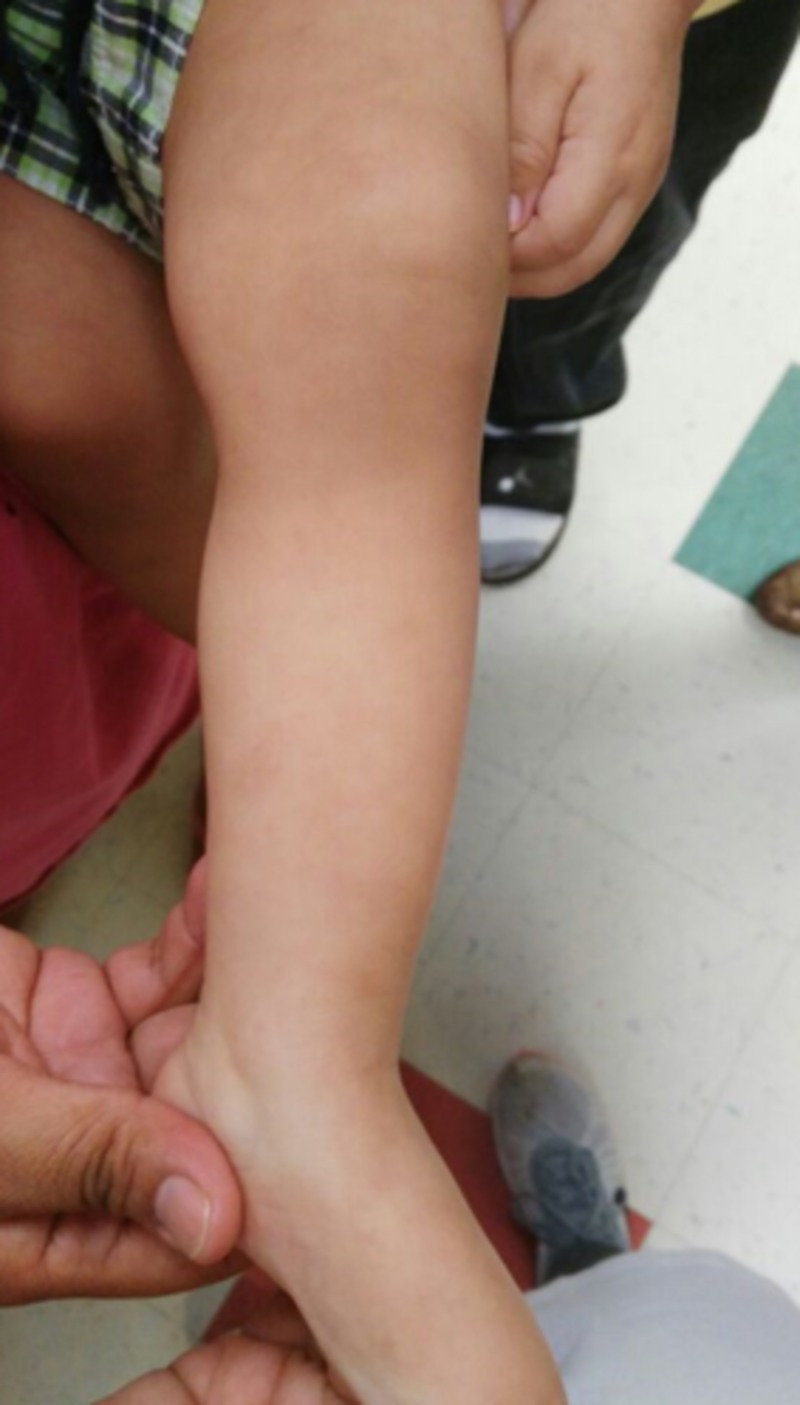
Patient after four days treated with 5 mg Loratadine and baking soda baths

The lesions were compatible with contact dermatitis caused by a caterpillar (lepidopterism). The patient was recommended to take loratadine 5 mg daily for five days and was advised to bathe the area with water and baking soda and return to the clinic once therapy was finished. After four days, the patient returned to the clinic and the rash had completely resolved. All medications were subsequently discontinued (Figure 3).

## Discussion

We presented a case of lepidopterism where the parents brought in the specimen that caused the cutaneous reaction, therefore the diagnosis was clear but other differential diagnoses should be taken into consideration in the clinical setting.

Generally, episodes of lepidopterism are isolated, however, certain people may be more prone to exposure with the moths. Particularly, in rural areas, workers in farms and gardens have an increased exposure, while in the cities and urban areas, people who visit parks and areas with trees are exposed to these moths [7,8]. Exposure is typically increased during summer months, because people are out more often while it also coincides with a greater number of caterpillars due to their breeding season [9]. The other leading cause of contact is children touching or grabbing the caterpillars due to their harmless and fluffy appearance.

The patient was treated according to other case reports in the literature and as a standard case of suspected contact dermatitis. Although caterpillar stings are not an everyday diagnosis, the physician must always think about this possibility if a history of a patient being in parks, fields and open areas with trees is mentioned. One should always ask the patient or the patient's guardians if they saw the offending animal or agent and encourage to describe it. In the next few paragraphs, we’ll review the most important aspects of the pathology, including presentation, diagnosis and treatment.

Clinical presentation

The clinical presentation varies depending on the type of moth, hairy coat and venom. A specific clinical history is crucial for diagnosis, as there are no tests to confirm this diagnosis. The clinician must keep in mind the various possible etiologies of contact dermatitis, including insects [10]. Common symptoms include intense pain minutes after the sting, vomiting, nausea and abdominal pain. The main clinical manifestation is an erythematous maculopapular rash, that can last up to five days. There is a description of a classic "tram-track" pattern of hemorrhagic papules, which is sometimes helpful to diagnose Megalopyge stings [11-13]. Rarely, lepidopterism cases may be severe enough for the patient to present with systemic symptoms, including anaphylactic shock, however, there has not been a reported case from this specific caterpillar species [14].

Differential diagnoses

Differential diagnoses may include allergic contact dermatitis, atopic dermatitis, scabies (which can be ruled out with the location of the skin lesions and the absence of burrows), other insect or animal bites or polymorphous light eruption. Furthermore, systemic symptoms of asthma or other atopic signs must be screened for to exclude an allergic pathology as a diagnosis [15].

Treatment

The condition is usually self-limited, and treatment focuses mainly on symptomatic relief. Ice or other measures to reduce pain may be utilized and corticosteroid creams are useful for reducing the rash in the acute setting but should not be used for a prolonged time due to their local adverse effects. Preferably, second generation antihistamines should be prescribed to reduce pruritus. Typically, nonsteroidal anti-inflammatory drugs (NSAIDs) are ineffective to reduce pain. Bathing the affected area with baking soda can reduce the inflammation and burning sensation, and thorough washing of the rash with soap and water is useful to reduce possible remains of the caterpillar's venom, as spines may remain embedded in skin and worsen the contact dermatitis. If that is the case, removal of the remaining spines is recommended, either manually with forceps or with adhesive tape [16]. It is important to educate parents or guardians on the possible complications of the reaction, and advise to closely observe the resolution of rash while also be aware for worsening symptoms.

## Conclusions

In the clinical setting, when encountering an acute onset cutaneous reaction, especially in pediatric population, it is important to obtain a complete history from the parents or guardians, including recent exposure and trips to parks or forests, since this factor can affect the differential diagnosis and broaden it. Also, physicians must educate parents to be alert while on trips to these places, especially in the summer months, and recommend their children to avoid grabbing insects or animals. If contact is made with the hair or spines of these insects, prompt bathing and washing the area with soap and water must be performed to avoid further contact and the ensuing cutaneous reaction.
